# Adipose Tissue-Derived Mesenchymal Stem Cells Have a Heterogenic Cytokine Secretion Profile

**DOI:** 10.1155/2017/4960831

**Published:** 2017-05-31

**Authors:** Yongkang Wu, Martin J. Hoogduijn, Carla C. Baan, Sander S. Korevaar, Ronella de Kuiper, Lin Yan, Lanlan Wang, Nicole M. van Besouw

**Affiliations:** ^1^Nephrology & Transplantation, Department of Internal Medicine, Erasmus MC, University Medical Center Rotterdam, Rotterdam, Netherlands; ^2^West China Hospital, Department of Laboratory Medicine, Sichuan University, Chengdu, China

## Abstract

Mesenchymal stem cells derived from adipose tissue (ASC) have immune regulatory function, which makes them interesting candidates for cellular therapy. ASC cultures are however heterogeneous in phenotype. It is unclear whether all ASC contribute equally to immunomodulatory processes. ASC are also responsive to cytokine stimulation, which may affect the ratio between more and less potent ASC populations. In the present study, we determined IL-6 receptor (CD126 and CD130 subunits) and IFN-*γ* receptor (CD119) expression on ASC by flow cytometry. The production of IL-6 and IFN-*γ* was measured by ELISA and the frequency of IL-6 and IFN-*γ* secreting cells by ELISPOT. The results showed that ASC did not express CD126, and only 10–20% of ASC expressed CD130 on their surface, whereas 18–31% of ASC expressed CD119. ASC produced high levels of IL-6 and 100% of ASC were capable of secreting IL-6. Stimulation by IFN-*γ* or TGF-*β* had no effect on IL-6 secretion by ASC. IFN-*γ* was produced by only 1.4% of ASC, and TGF-*β* significantly increased the frequency to 2.7%. These results demonstrate that ASC cultures are heterogeneous in their cytokine secretion and receptor expression profiles. This knowledge can be employed for selection of potent, cytokine-producing, or responsive ASC subsets for cellular immunotherapy.

## 1. Introduction

Mesenchymal stem cells (MSC) are adult stem cells with the ability to differentiate into several lineages, such as osteoblasts, chondrocytes, myocytes, and adipocytes [1]. Initial studies focused on MSC derived from the bone marrow but subsequently the presence of MSC in, amongst others, adipose tissue was demonstrated [2], so-called adipose tissue-derived mesenchymal stem cells (ASC). Adipose tissue has some advantages above the bone marrow as a source of MSC as it is relatively easy to access, it is abundant, and the procedure for isolating ASC is easy [3]. It is well known that ASC have a broad immune regulatory function [4, 5], which makes them suitable for cellular therapy.

ASC suppress the proliferation and inflammatory cytokine production of activated immune cells and induce the formation of immunoregulatory cell types, such as regulatory T cells and alternatively activated macrophages [6, 7]. Bone marrow and adipose tissue-derived MSC employ similar mechanisms for immunomodulation [8]. These include targeting immune cells via both cell contact-dependent and cell contact-soluble interactions, such as via the inhibitory costimulatory programmed death ligand 1 (PD-L1) pathway [9] and via the secretion of soluble factors [10]. A multitude of factors have been proposed to play a role in the immunomodulatory effect of MSC, including hepatocyte growth factor (HGF) [11], HLA-G [10], and IL-6 [12]. Although generally seen as a proinflammatory cytokine, IL-6 has a clear dual function and can enforce as well as suppress immune responses, depending on the conditions [13]. Furthermore, the tryptophan-depleting enzyme indoleamine 2,3-dioxygenase (IDO) plays a major contribution to the antiproliferative effect of MSC [14]. MSC in their turn respond to inflammatory cytokines, in particular IFN-*γ*, but also TNF-*α* and IL-17, by dramatically increasing IDO and PD-L1 expression thereby strongly enhancing their immunosuppressive properties [15–17]. MSC are potent secretors of anti-inflammatory TGF-*β* which contributes to their immune regulatory effects [18], and in addition, TGF-*β* affects the immune regulatory function of MSC themselves too [19].

Although MSC are clearly involved in cross talk with immune cells, not all MSC may do this in the same way as there is considerable heterogeneity within MSC populations. There is heterogeneity in the differentiation potential of MSC [20, 21], and there is heterogeneity in the expression of cell surface markers such as STRO-1 and CD271, and STRO-1 and CD271 expressing MSC have been demonstrated to possess enhanced immunomodulatory capacity [22, 23]. The heterogeneity of MSC may impair their therapeutic efficacy and introduce variations between studies [24]. One the other hand, it offers opportunities to isolate super-potent MSC from heterogenic populations.

ELISPOT assays are widely used for nonadherent lymphocytes and is more sensitive than ELISA. The ELISPOT assay had not been described for adherent ASC. The advantage of ELISPOT is the sensitive technique for detecting a cytokine at single cell level and allowing frequency analysis [25–27].

We questioned whether ASC populations are heterogenic in their cytokine secretion and cytokine receptor expression profile. Furthermore, we examined whether potential heterogeneity was affected by cytokine stimulation of ASC. In the present study, we enumerate IL-6 and IFN-*γ* in single-secreting ASC by ELISPOT assay. Furthermore, we studied the frequency of IL-6 and IFN-*γ* receptor expressing ASC and examined the effect of IFN-*γ* and TGF-*β* stimulation on ASC cytokine production.

## 2. Materials and Methods

### 2.1. Isolation and Culture of ASC

ASC were isolated from human subcutaneous adipose tissue that became available upon donation of living kidney donors after written informed consent (protocol number MEC-2006-190 approved by the Medical Ethics Committee of the Erasmus MC, Rotterdam) as previously described [28]. In brief, after mechanical disruption and enzymatic digestion of the adipose tissue, the cells were collected in minimum essential medium-*α* (MEM-*α*) (Sigma-Aldrich, St. Louis, MO); supplemented with 1% penicillin/streptomycin solution (P/S; 100 IU/mL penicillin, 100 IU/mL streptomycin; Lonza, Verviers, Belgium), 2 mM L-glutamine (Lonza), and 15% fetal bovine serum (FBS; Lonza) (MSC medium); and seeded in T175 culture flasks (Greiner Bio-One, Kremsmunster, Germany) at 37°C, 5% CO_2_, and 95% humidity. Cultures were refreshed twice weekly. When the cultures reached 90% confluence, ASC were removed from the culture flasks using 0.05% trypsin-EDTA (Life Technologies, Bleiswijk, Netherlands). ASC were used for experiments between passages 1 and 5.

### 2.2. Flow Cytometric Analysis

ASC were immunophenotypically characterized by staining for CD45-FITC, CD31-FITC, CD13-PECy7, CD73-PE, and CD90-APC (all BD Biosciences, San Jose, CA). For detection of IL-6 and IFN-*γ* receptors, 400,000 ASC (*n* = 3) were stained for two IL-6 receptor subunits (CD126 and CD130) and IFN-*γ* receptor (CD119). The cells were incubated with anti-CD126-PECy7 (BioLegend, San Diego, CA), anti-CD130-BV421 (BD Biosciences, San Jose, CA), anti-CD119-APC (SB Sino Biological Inc., Beijing, China), or isotype-matched control antibodies (eBioscience, San Diego, CA) in the dark for 30 min at room temperature. Thereafter, the cells were washed twice with FACSFlow (BD Biosciences) and measured on a FACS Canto II flow cytometer (BD Biosciences) and analyzed with Kaluza Analysis 1.3 software (Beckman-Coulter, Brea, CA).

### 2.3. Stimulation of ASC

ASC were stimulated for 72 hours with 50 ng/mL IFN-*γ* (Life Technologies, USA) or 10 ng/mL TGF-*β* (Peprotech, USA) prior to experiments in MEM-*α* with P/S, 2 mM L-glutamine, and 15% FBS. Unstimulated control cells were cultured in parallel.

### 2.4. IL-6 and IFN-*γ* ELISPOT Assay

PVDF membrane-bottomed 96-wells plates (multiscreen, Millipore Ireland) were incubated with 70% ethanol for 1 minute at room temperature. After washing the wells with PBS, the wells were precoated with anti-IL-6 mAb or anti-IFN-*γ* mAb (U-CyTech Biosciences, Utrecht, Netherlands) and blocked with PBS containing 1% BSA according to the manufacturer's protocol. In brief, ASC (*n* = 4: unstimulated, IFN-*γ* stimulated, and TGF-*β* stimulated) were seeded in triplicate at a concentration of 4000, 2000, 1000, 500, 250, 125, 62.5, and 31.25 ASC per well. Cells were incubated for 24 hours at the coated ELISPOT plate at 37°C, 5% CO_2_, and 95% humidity to allow spot formation to occur. After incubation, the cells were lysed with ice-cold milli-Q water and the plates washed extensively. Subsequently, the wells were incubated with a biotinylated goat antihuman IL-6 or antihuman IFN-*γ* polyclonal Ab (U-CyTech Biosciences) for 1 hour at 37°C. After washing the wells, IL-6 spots were detected by streptavidin-HRP conjugate and an AEC substrate for IL-6 (U-Cytech Biosciences). IFN-*γ* spots were detected with phi-labeled goat antibiotin Ab (U-Cytech Biosciences) and a reagent that activates phi (reagent I + II, U-Cytech Biosciences). The reactions were stopped when spots were visualized by adding milli-Q water to the wells. The spots were counted by Bioreader 6000 Elispot-reader (BioSys GmbH, Karben, Germany).

### 2.5. ELISA

After pretreating ASC (*n* = 5) with IFN-*γ* or TGF-*β* for 72 hours, the cells were trypsinised and seeded at 50 (IL-6 ELISA) and 4000 (IFN-*γ* ELISA) cells per well in 96-well plates (Greiner Bio-One, Kremsmünster, Austria). After 24 hours, conditioned medium was collected. The production of IL-6 and IFN-*γ* was determined using ELISA kits (U-CyTech Biosciences, Utrecht, Netherlands) according to the manufacturer's instructions.

### 2.6. Statistical Analysis

The effect of ASC pretreated with IFN-*γ* or TGF-*β* were analyzed by one-way ANOVA to compare differences in number of cytokine producing IFN-*γ* and IL-6 producing ASC and by two-tailed paired *t*-test to compare differences in IFN-*γ* and IL-6 ELISA. *p* values  < 0.05 were considered significant.

## 3. Results

### 3.1. Immunophenotype of ASC

**Figure 1:**
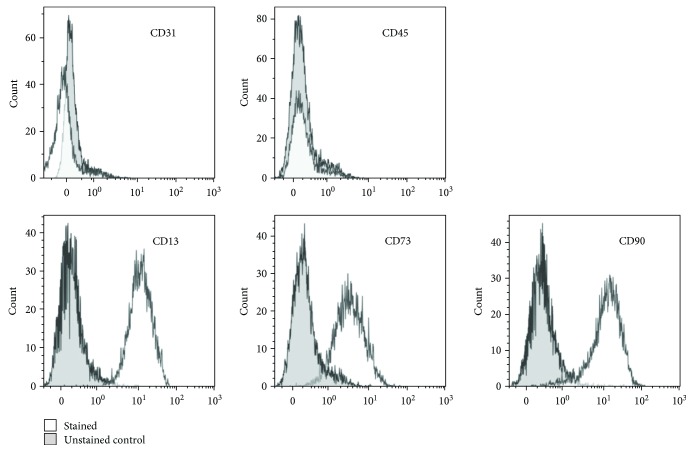
Immunophenotype of ASC. Flow cytometric analysis of the immunophenotype of ASC, demonstrating a lack of CD31 and CD45 expression and positive expression of CD13, CD73, and CD90.

ASC showed a typical spindle-shaped morphology (data not shown) and lacked expression of the hematopoietic cell marker CD45 and of the endothelial cell marker CD31 (Figure 1). Nearly all cells expressed the markers CD13, CD73, and CD90, confirming the ASC phenotype of the cells.

### 3.2. Heterogeneity in ASC Cytokine Secretion Profiles

**Figure 2:**
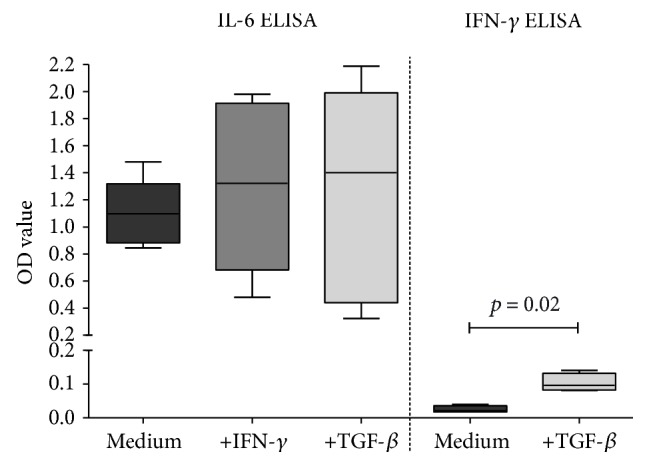
Secretion of IL-6 and IFN-*γ* by ASC. ASC (*n* = 5) (50 ASC for IL-6, 4000 ASC for IFN-*γ*) were cultured without or in the presence of IFN-*γ* or TGF-*β* for 72 h and washed and reseeded. IL-6 and IFN-*γ* ELISA were performed in 20 h conditioned medium. Data is presented as box and whisker plot (median and range). More IFN-*γ* was produced after pretreatment with TGF-*β* (*p* = 0.02, two-tailed paired *t*-test).

To determine whether control ASC and ASC pretreated with IFN-*γ* or TGF-*β* were capable of secreting IL-6 and IFN-*γ*, ELISA were performed on conditioned medium samples. IL-6 was detectable in a conditioned medium from 96-well plates containing as few as 50 ASC per well, demonstrating that IL-6 was abundantly secreted by ASC. There was no difference between control ASC and IFN-*γ*- or TGF-*β*-pretreated cultures in the level of IL-6 secretion (Figure 2). IFN-*γ* secretion by 4000 ASC was hardly detectable. TGF-*β* treatment of MSC significantly increased IFN-*γ* secretion (mean ± SD, OD 0.0248 ± 0.0102 versus 0.1035 ± 0.0268; *p* = 0.02: Figure 2).

**Figure 3:**
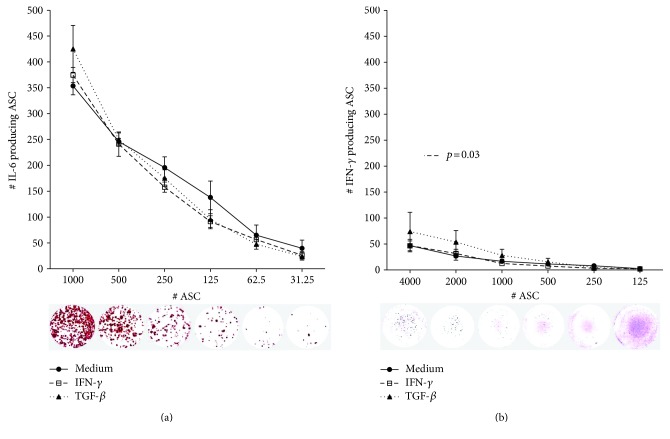
Frequency of IL-6 and IFN-*γ* secreting ASC. ASC (*n* = 4) were cultured without or in the presence of IFN-*γ* or TGF-*β* for 72 h and washed and reseeded at different cell densities. Frequencies of IL-6 (a) and IFN-*γ* (b) secreting ASC were determined by ELISPOT assay. Representative examples of the ELISPOT assay are shown. After TGF-*β* treatment, a higher frequency of IFN-*γ* producing ASC was found (*p* = 0.03, one-way ANOVA).

To examine whether IL-6 is secreted by all ASC, or whether a subpopulation of the cells is responsible for the IL-6 levels found, ELISPOT assay was performed. ASC were seeded in 96-well plates at different densities, ranging from 31 to 1000 cells per well to determine the frequency of IL-6 producing cells. At the lower ranges (31–125 ASC), we found approximately one IL-6 spot per ASC seeded (Figure 3(a)). At higher ASC ranges, the relative number of spots declined to less than 400 spots per 1000 ASC seeded. This is probably due to the crowding of spots at higher ASC numbers, which will start to overlap and subsequently be read as a single spot [29]. Pretreatment of ASC with IFN-*γ* or TGF-*β* had no effect on the frequency of IL-6 producing ASC. Although IFN-*γ* secretion by ASC was hardly detectable by ELISA, we detected a frequency of 1.2%, 1.4%, and 1.7% of IFN-*γ* secreting ASC, detectable for the 4000, 2000, and 1000 seeded ASC, respectively (Figure 3(b)). After TGF-*β* treatment, a significantly higher frequency of IFN-*γ* producing ASC were found; 1.9%, 2.7%, and 2.8%, respectively (*p* = 0.03). These results demonstrate that whereas all ASC secrete IL-6, a small subpopulation secretes IFN-*γ*, thereby demonstrating heterogeneity in ASC cultures concerning cytokine secretion.

### 3.3. Heterogeneity in ASC Cytokine Receptor Expression

**Figure 4:**
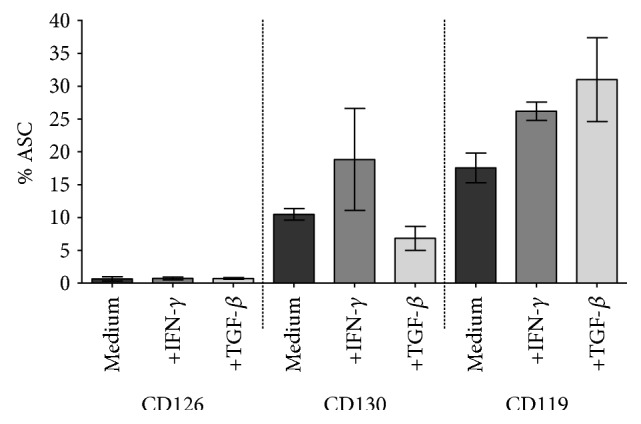
Expression of IL-6 receptor and IFN-*γ* receptor on ASC. ASC (*n* = 3) were cultured without or in the presence of IFN-*γ* or TGF-*β* for 72 h and trypsinised and analyzed by flow cytometry. The IL-6 receptor subunit CD126 was not detected, and CD130 was detected in a small percentage of the ASC. The IFN-*γ* receptor (CD119) was detected on a subpopulation of ASC. Presented as mean and SEM.

IL-6 secreted by ASC may have a paracrine and/or an autocrine function. To examine whether ASC-secreted IL-6 has an autocrine function, we measured the expression of the IL-6 receptor *α*-chain (CD126) and the IL-6 receptor *β*-chain (CD130) by flow cytometry (Figure 4). CD126 was not detected on the surface of ASC. A subpopulation (10%) of ASC expressed CD130, and pretreatment with IFN-*γ* resulted in an increased expression of CD130 to 18% of ASC (Figure 4). However, the absence of CD126 indicates that no functional IL-6 receptors are present on ASC. We also measured the expression of the IFN-*γ* receptor 1 (CD119). CD119 was expressed on a subpopulation of 18% of ASC. Pretreatment with IFN-*γ* or TGF-*β* resulted in a higher percentage of CD119 positive ASC, 26% and 31%, respectively (Figure 4).

## 4. Discussion

MSC are found in all tissues [30] and in the search of finding the most assessable, best expandable, and most effective MSC type for therapy, remarkable similarities concerning surface antigen expression, immunosuppressive activity, and differentiation ability between MSC of different tissue sources have been observed [3, 31]. There are, however, subtle differences between MSC of different sources, such as in levels of chemokine receptor expression and paracrine factor production, and in the resistance to apoptosis, which may reflect different therapeutic efficacy [32–34].

Even within populations of MSC from one tissue source subpopulations of MSC can be identified. For instance, LNGFR^+^THY-1^+^VCAM-1^hi+^ MSC have been identified as a population of MSC with enhanced clonogenic properties [35]. James et al. have demonstrated that bone marrow MSC contain distinct immunomodulatory and differentiation-competent subtypes [36] and, for example, STRO-1-enriched MSC display a more suppressive effect on lymphocyte proliferation than MSC [22]. Data from the present study suggests that within populations of ASC there is variation in the immunoregulatory function of ASC. A small fraction of ASC produced IFN-*γ*, and a subpopulation of ASC expressed the IFN-*γ* receptor, which distinguishes these cells from other ASC with respect to their response to inflammatory conditions where IFN-*γ* is around. It is very likely that similar selective expression patterns are found for other receptors and soluble factors. We found no selective secretion of IL-6. All ASC secreted IL-6 at single cell level whereas no expression of the IL-6 receptor *α*-chain was found on ASC and only 10% expressed the IL-6 receptor *β*-chain. This demonstrates that IL-6 secreted by ASC has a paracrine signaling role. This has been demonstrated in studies showing that IL-6 secreted by MSC plays a role in the regulation of monocytes [37] and dendritic cells [38].

It is well established that ASC change their immunomodulatory function after exposure to cytokines. It is therefore surprising that the secretion of IFN-*γ* and expression of IFN-*γ* receptor or IL-6 receptor was only slightly affected by pretreatment of the ASC with IFN-*γ* or TGF-*β*. We furthermore found no changes in the level of IL-6 secretion, while significantly more IFN-*γ* was produced after pretreatment with TGF-*β*. This effect of TGF-*β* was also found in the IFN-*γ* ELISPOT. It is possible that pretreatment with IFN-*γ* or TGF-*β* affects the secretion by ASC of other cytokines that were not examined in the present study or that pretreatment with other cytokines has a more profound effect on ASC. Ageing of ASC in culture may be another factor influencing the function of ASC. We recently demonstrated that the MSC phenotype remains stable until passage 12 and that the immunosuppressive capacity of MSC was reduced from passage 8 onwards [39]. In the present study, ASC between passages 1–5 were used. At these passages, there is no evidence of effects on the phenotype and function of MSC.

This preliminary study demonstrates to our knowledge for the first time that the ELISPOT assay can be used to determine the heterogeneity of MSC with respect to their cytokine secretion. Although MSC are adherent cells and thus physically block patches of the ELISPOT plates, the cytokines secreted by MSC do form spots that are detectable after lysis of the MSC. At high-seeding densities, it was observed that the frequency of IL-6 secreting ASC was decreased. This could have a biological origin stemming from an inhibitory effect of MSC on their neighbors' cytokine secretion. Alternatively, the reason for this observation may be that spots start to overlap at high cell densities leading to an underestimation of spots. It is therefore important to take different seeding densities of MSC in consideration.

## 5. Conclusions

The ASC population is heterogenic in their cytokine secretion and cytokine receptor expression profile. Determining the frequency of cytokine or growth factor producing ASC by ELISPOT assay is a useful novel tool in the characterization of (clinical) ASC batches that can be used as a potency assay.
